# Electroacupuncture on Hemifacial Spasm and Temporomandibular Joint Pain Co-Morbidity: A Case Report

**DOI:** 10.3389/fneur.2022.931412

**Published:** 2022-06-28

**Authors:** Jian-peng Huang, Zhan-mou Liang, Qi-wen Zou, Jie Zhan, Wen-ting Li, Sheng Li, Kai Li, Wen-bin Fu, Jian-hua Liu

**Affiliations:** ^1^Research Team for Acupuncture Effect and Mechanism, The Second Affiliated Hospital of Guangzhou University of Chinese Medicine, Guangzhou, China; ^2^Clinical Medical College of Acupuncture Moxibustion and Rehabilitation, Guangzhou University of Chinese Medicine, Guangzhou, China; ^3^Department of Otorhinolaryngology, The Second Affiliated Hospital of Guangzhou University of Chinese Medicine, Guangzhou, China

**Keywords:** electroacupuncture, hemifacial spasm, temporomandibular joint pain, co-morbidity, case report

## Abstract

Hemifacial spasm (HFS) and temporomandibular joint (TMJ) pain are common facial diseases which cause depression, anxiety, insomnia, and poor quality of life. However, currently there are still no effective therapies to treat HFS and TMJ. Electroacupuncture (EA) has advantages of safety, rapid work, easy operation and convenience. Here, we reported a case of a 50-year-old woman who presented with irregular spasm of eyelids and facial muscles on the left side, and TMJ pain on the right side. The patient had been treated with carbamazepine (20mg per day) and alternative therapies for a year, but still not much improvement in the symptoms. The scores of the Jankovic Rating Scale (JRS), global rating scale (GRS), and visual analog scale (VAS) were 7, 60, and 7 points, respectively. The EMG test showed that the spastic side had higher R1 amplitude, longer R2 duration, and larger R2 area than the non-spasmodic side, and the occurrence rate of the lateral spread responses (LSR) in the Orbicularis oris and the Orbicularis oculi muscle was 60% and 40%, respectively. We considered this patient had left HFS and right TMJ pain. EA was successfully undertaken for two periods over 30 weeks. After EA, JRS and VAS were reduced sharply, and the symptoms of HFS were stable without recurrence. However, the frequency of the lower eyelid increased gradually during the 6-month follow-up. These findings reveal that EA with the frequency of 2 Hz and intensity of ~ 1–2 mA may be a benefit for alleviating symptoms of HFS and TMJ pain without adverse reaction. The potential mechanisms of EA in HFS and TMJ pain co-morbidity involve brain stem mechanism and DNIC mechanism for distal acupuncture and segmental mechanism for local acupuncture analgesia.

## Highlights:

- A total of 30 weeks of EA has benefits for relieving the spasm of HFS and reducing the pain of TMJ, and lasts for 3 months.
- Sensory input from the hand may be *via* the medial lemniscus, then inhibiting afferent from the face in the trigeminal nucleus (TN).
- Diffuse noxious inhibitory controls (DNIC) and segmental mechanisms play an important role in acupuncture analgesia effect.

## Introduction

Hemifacial spasm (HFS) is a frequent disorder characterized by involuntary contractions of those muscles innervated by the facial nerve on one side of the face. The symptoms can appear as tonic or clonic and intermittent or permanent. The incidence rate of HFS was about 1 per 10,000 people (1). To date, the gold standard for the diagnosis of HFS is still lacking. Clinicians diagnose mainly based on clinical symptoms. HFS can cause depression, anxiety, insomnia, poor quality of life, and so on (2, 3).

At present, clinicians mainly use drugs, surgery, and other methods to treat HFS; however, the effects of these therapies are relatively limited and accompanied by some adverse reactions. For example, carbamazepine and clonazepam may be effective in some mild patients, but there are some side reactions, such as drowsiness, headaches, and dizziness (4). The botulinum toxin type A is another therapy to reduce the symptoms of HFS, but its effect can only last up to 3–6 months and long-term use requires increased doses (5, 6). Previous studies reported that botulinum toxin type A can paralyze facial nerves and cause artificial facial paralysis (7, 8), leading to a main long-term side effect of facial asymmetry (9). Surgery (e.g., facial nerve decompression and microvascular decompression) is the most commonly used method for radical HFS, but always rejected by patients due to the adverse effects, such as facial palsy and transientor permanent cranial nerve deficits (10, 11).

Temporomandibular joint (TMJ) disorder is the most common disease of the oral and maxillofacial region, of which the main clinical manifestations are pain in the TMJ, joint snapping during exercise, mandibular movement disorder, ear pain, tinnitus, dizziness, neck pain, and headache (12, 13). It affects ~15%−20% of the population (14). Previous MRI-based studies have demonstrated that TMJ pain is associated with multiple factors including joint effusion, bone marrow edema, and osteoarthritis (15, 16). The treatment protocols for TMJ disorders vary over the years (17). Pharmacotherapies were used to reduce pain and improve function, such as anti-inflammatory drugs, muscle relaxants, and Botulinum toxin, for mild to moderate TMJ disorder (18). Moreover, in terms of surgical treatment, arthroscopic therapy is more popular because of its higher success rate (17).

Electroacupuncture (EA) is a combination of conventional acupuncture with electrical stimulation on acupoints, which can enhance the sensory input from peripheral system (19). EA has the advantages of safety, rapid work, easy operation, and convenience, which has been widely used in the management of neurological and arthrosis diseases (20, 21). We report a case of HFS and TMJ pain co-morbidity treated successfully by the peripheral EA. This study was approved by the Ethics committee of the Guangdong Provincial Hospital of Chinese Medicine (AF/04-07.0/10.0).

## Case Description

A 50-year-old female patient, who had suffered from hemifacial spasm (HFS) for nearly 6 years, came to the acupuncture clinic on 16 February 2021. She complained of irregular spasms of the eyelids and facial muscles on the left side, and the occurrence of TMJ pain on the right side, accompanied with tinnitus, dizziness, neck pain, and headache, which seriously affected her health and daily life. Before her first visit to our hospital, she was diagnosed with primary HFS in another hospital in 2015. In the following 5 years, her symptoms worsened when she was stressed, tired, and fatigued, with increased frequency and severity of intermittent twitching of the left lower eyelid and lower face. Hereafter, she had been treated with carbamazepine (20 mg per day) and alternative therapies (e.g., Chinese herbal medicine, moxibustion, Guasha therapy, facial massage, etc.) for a year, but still not much improvement in the symptoms.

Physical examination showed that there was obvious twitching of the left facial muscles, the eyes could not be opened due to spasms on the upper face, the degree of spasm on the lower face was less than that on the upper face, the facial muscles on both sides were not symmetrical, there were tenderness and clicking sounds at the right temporomandibular joint without redness and swelling, and no joint deformation. Her Jankovic Rating Scale (JRS) was 7 points, 4 points for the severity and 3 points for the frequency, and her global rating scale (GRS) and visual analog scale (VAS) were 60 points and 7 points, respectively.

**Figure 1:**
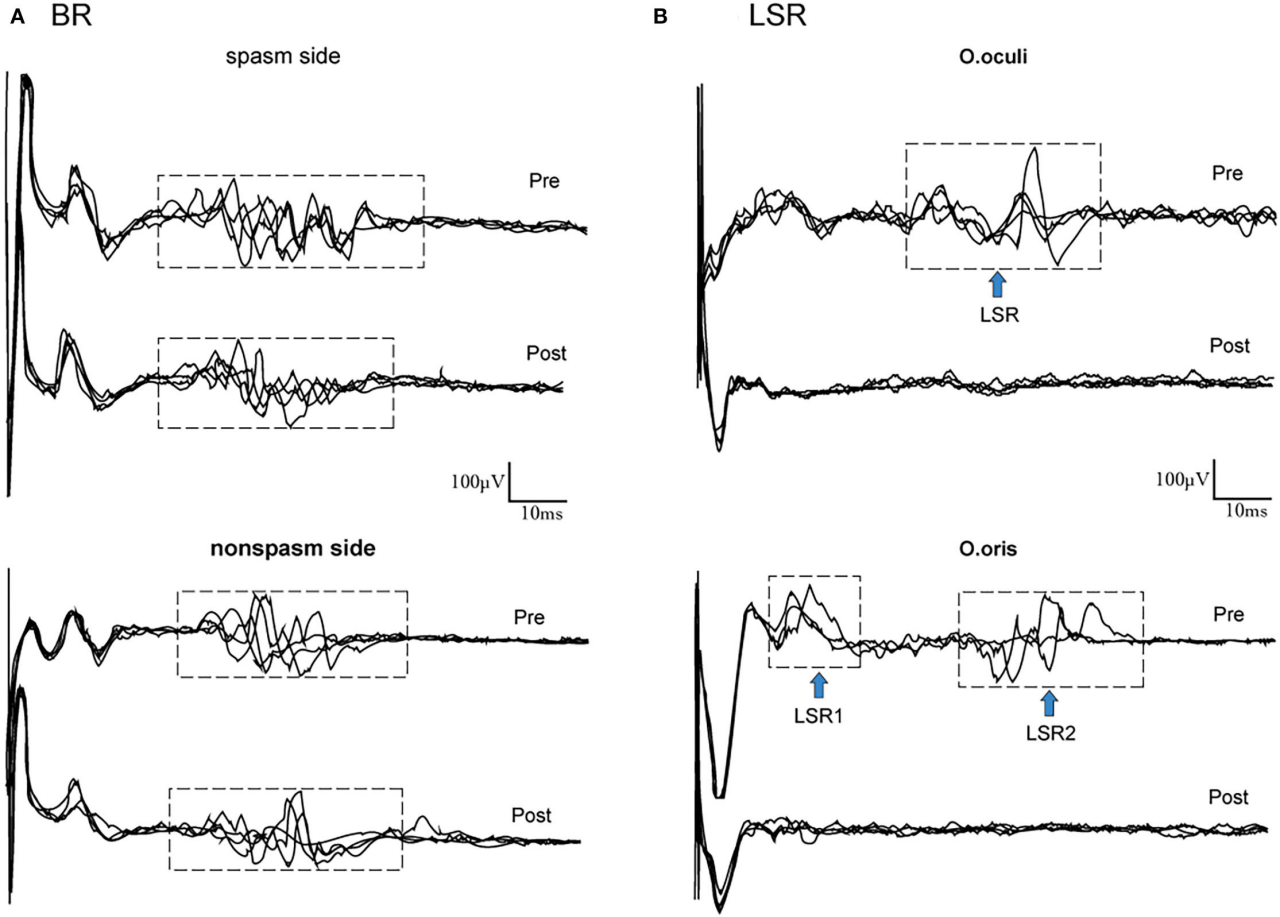
Raw data of BR and LSR. **(A)** Changes in BR. Compared to nonspasm side at baseline, the amplitude of R1 was higher, the duration of R2 was longer, and the area of R2 was larger in the spasm side. After 30 weeks of EA treatment, the duration of R2 was shortened and the area of R2 was reduced in the spasm side, and there were no changes in the nonspasm side. **(B)** Occurrence of LSR in O.oc and O.or (the blue arrow). After EA, no LSR was found in the facial muscles. BR, blink reflex; LSR, lateral spread responses; O.oc, Orbicularis oculi muscle; O.or, Orbicularis oris muscle.

Considering the recurrent symptoms and long course of HFS, we conducted relevant detection of the facial nerve, including blink reflex (BR) and lateral spread responses (LSR). We recorded the EMG activities in the Orbicularis oris (O.or) or the Orbicularis oculi (O.oc) muscle by stimulating the mandibular or zygomatic branches of the facial nerve, respectively. EMG test showed that the spastic side had higher R1 amplitude, longer R2 duration, and larger R2 area than the non-spasmodic side (Figure 1A, Supplementary Table S1). In ten stimuli, the occurrence rate of the LSR in O.oc was 60% and in O.or was 40%.

**Figure 2:**
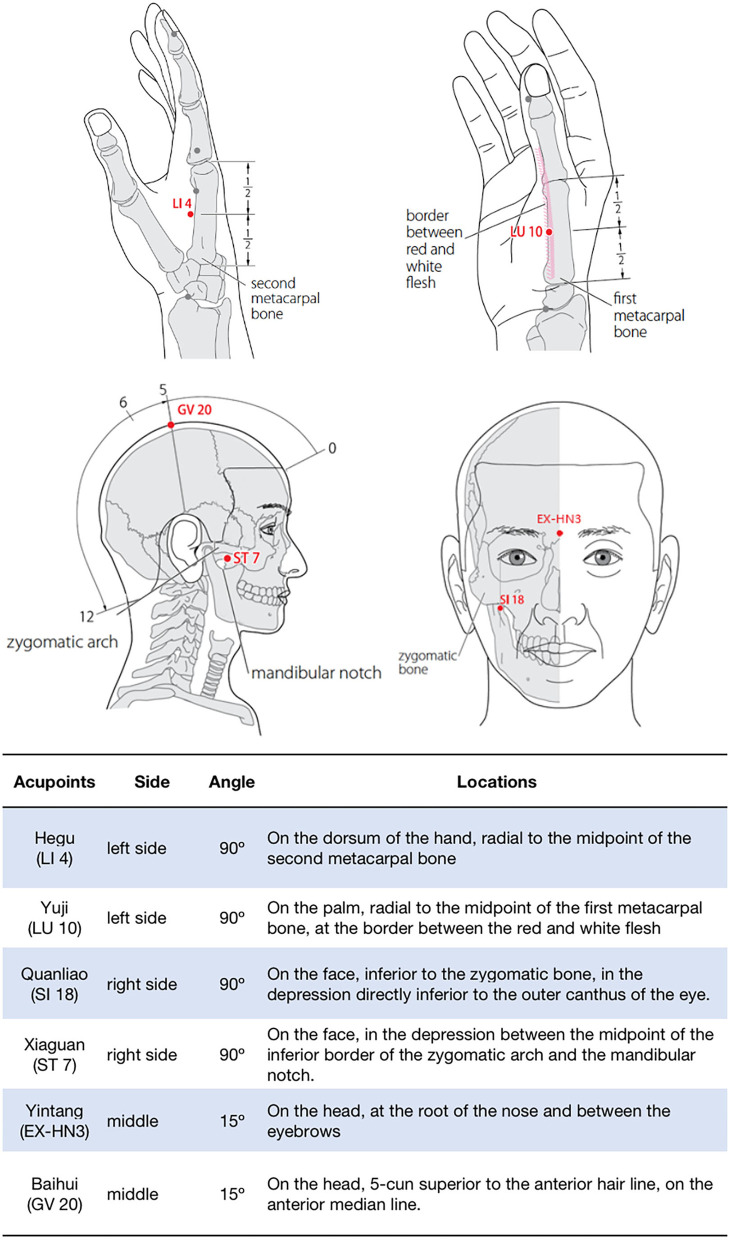
Locations of acupoints. (WHO (22)).

We considered this patient had left HFS and right TMJ pain based on the symptoms, physical examination, and EMG test. The onset stages of co-morbidity were severe. Her main pathological mechanism may be related to the hyperexcitability of the facial motor nucleus. Given her previous treatment protocols and her refusal to take medicines, we used EA to reduce her symptoms. According to the classical TCM theory “the treatment of ora-facial diseases by acupuncture at Hegu (LI4) acupoint,” which means Hegu acupoint can effectively treat the ora-facial diseases, such as toothache, facial paralysis, etc. So, we selected the following acupoints (22): LI 4 (Hegu), LU 10 (Yuji), SI 18 (Quanliao), ST 7 (Xiaguan), EX-HN 3 (Yintang), and GV 20 (Baihui). The locations of the above acupoints are shown in (Figure 2). Manipulate program: The patient is made to lie supine on the treatment bed and the acupuncturist stands on his right side to locate the acupoints. After skin disinfection, the acupuncturist inserts a stainless steel needle (0.25 × 25 mm, Hwato, Suzhou, China) to a 1.5-cm subcutaneous depth at an appropriate angle according to the acupoints. When the patient had a feeling of Deqi (namely soreness, numbness, warmth, heaviness, or distention around the acupoints), a constant-current (direct current). EA (HANS-200A, Nanjing, China) was applied to LI 4 and LU 10 (positive pole), SI 18 and ST 7 (positive pole), respectively. The frequency was 2 Hz and intensity was set at ~1–2 mA for 30 min, preferably with the muscle shivering mildly but without pain. The duration of EA was 30 min each session, two sessions a week, 20 sessions for the first 10 weeks, and then changed to 30 min each session, once a week, 20 sessions for the next 20 weeks. EA was performed by the same experienced acupuncturist registered in China. During treatment, no additional medications were used and no adverse events were found.

At the 10th week of treatment, the severity and frequency of the patient's facial spasms were relieved. The JRS was 4 scores (2 scores for severity and frequency, respectively), the GRS was 30 scores, and the VAS was 2 scores (Table 1). Also, she still felt mild tinnitus, dizziness, neck pain, and headache. By the 30th week of treatment, not only had her lower facial spasms gone, but her tinnitus, dizziness, neck pain, and headache had also reduced significantly. Meantime, her GRS and VAS were 20 and 0 score, respectively. However, she still had mild spasms on her lower eyelid. The JRS of her lower eyelid was 2 scores (1 score for severity and frequency, respectively; Table 1). The duration of R2 was shortened by 5.35ms, the area of R2 was reduced by 13.56%, and the LSR of O.oc and O.or disappeared on the spasm side (Figure 1B).

**Table 1:** Clinical assessments for HFS and TMJ pain at each time points.

**Outcomes**	**Before treatment (T0)**	**During treatment (T1)**	**After treatment (T2)**	**3-month follow-up (T3)**	**6-month follow-up (T4)**
JRS-Severity	4	2	1	1	1
JRS-Frequency	3	2	1	1	2
JRS total scores	7	4	2	2	3
GRS score	60	30	20	20	40
VAS score	7	2	0	1	1

**Figure 3:**
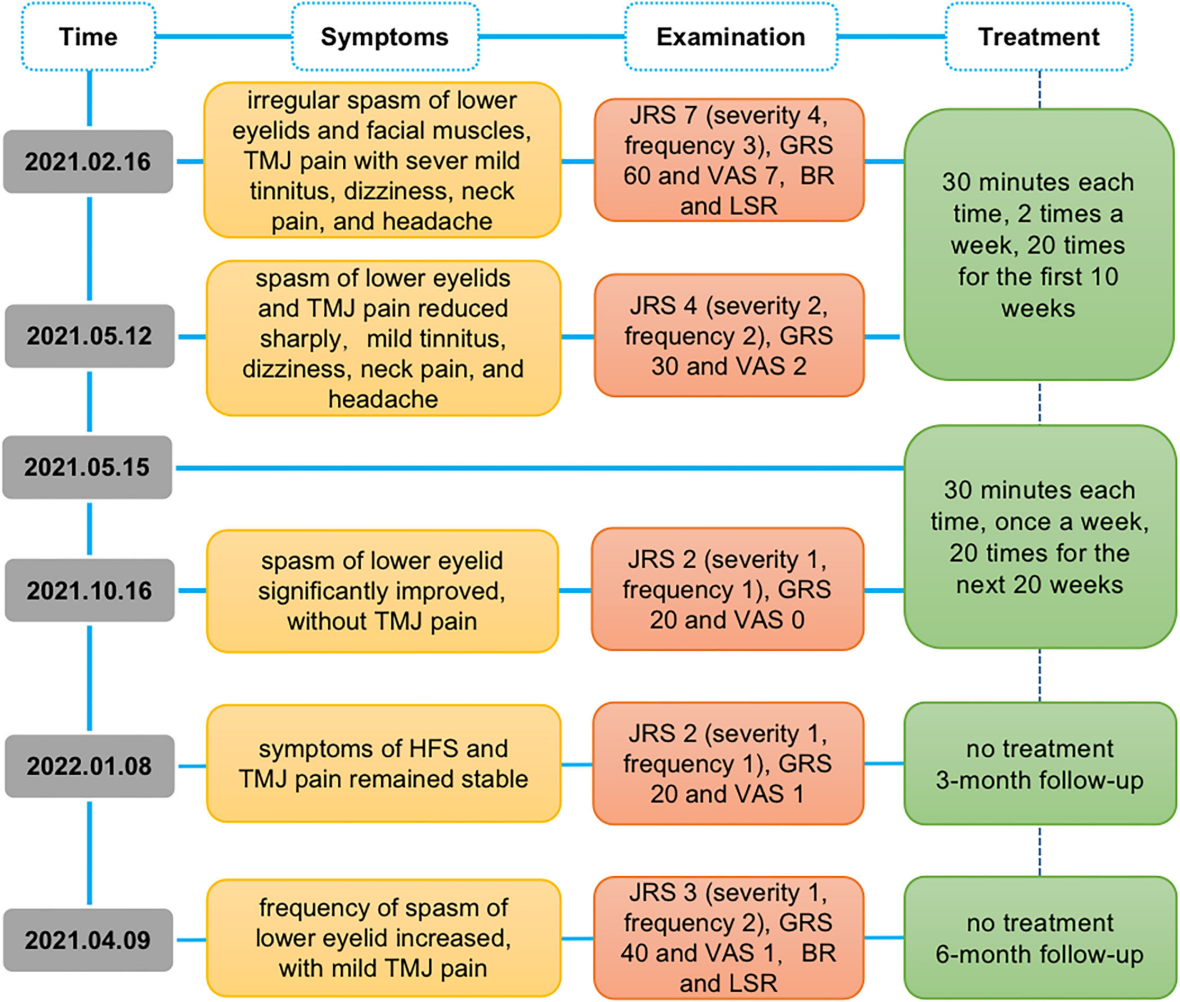
Study timeline. JRS, Jankovic Rating Scale; GRS, global rating scale; VAS, visual analog scale; BR, blink reflex; LSR, lateral spread responses.

Telephone follow-up was conducted in the 3rd month after the end of EA, and the symptoms of HFS were stable without recurrence, but the TMJ pain occurred intermittently. At the 6-month follow-up, the frequency of spasms (lower eyelid) increased gradually, but the severity remained stable (Table 1). The timeline of the intervention and outcomes is shown in (Figure 3.)

## Discussion

Electroacupuncture therapy is the method that adds an electrical stimulation pulse to acupuncture needles. It is safe and convenient. In this case, we showed that a peripheral electrical acupuncture stimulation can alleviate symptoms of HFS and TMJ pain, remaining stable for the following 3 months. Generally, in one way, it showed significant decreasing scores of JRS and GRS for HFS and VAS for TMJ pain, and in the other way, it declined the excitability of BR and LSR in spasm muscles.

For HFS, JRS and GRS are widely used for evaluating the symptoms (23, 24). The higher the scores, the more severe symptoms in the patient. The JRS is rated by a physician and is made up of two subscales, severity and frequency. Each of the subscale ranges from 0 to 4, where 0 indicates no symptoms and 4 indicates the most severe or frequent symptoms. GRS is a self-reported measure rated by the patient to show whether the symptoms improved or worsened after the treatment. In the beginning, the scores of the severity and frequency of spam showed a severe level, and after 30 weeks of EA treatment, the overall scores decreased, mild symptoms were found and kept stable at the 3rd month follow-up. At the 6th month follow-up, the frequency, but not the severity of spasms increased only in the eyelid, caused by the lack of sleep due to stress and anxiety.

Besides, we applied BR and LSR for a neurological test. BR is for the assessment of excitability changes at the brainstem inter-neuronal level, consisting of two components: R1 and R2. R1 is an oligo synaptic circuit from the principal trigeminal nucleus (PTN) to the facial nucleus, while R2 is mediated by a multi-synaptic circuit between the spinal trigeminal nucleus (STN) and facial nucleus with a chain of brainstem interneurons extending in the lateral reticular formation (25). After 30 weeks of EA treatment in the ipsilateral hand, the R1 component (amplitude) and the R2 component (duration and area) in the spasm side decreased, suggesting that the sensory input from the hand may inhibit that from the face in the trigeminal nucleus (TN). But no effect was found in the contralatral BR, indicating that the EA effect is mediated by the medial lemniscus. LSR (a facio-facial reflex), which is elicited by stimulating one branch of the facial nerve on the affected side, causing co-contraction of muscles innervated by other branches of the facial nerve (26), is an important monitoring tool for HFS, indicating hyperexcitability of the facial motor nucleus. The use of LSR is chosen for predicting the effectiveness of treatments, i.e., microvascular decompression (MVD) (27). In most of the studies, the occurrence rate of LSR is about 87% (28), and after MVD, the recovery rates have reached upto 90% (29). After 30 weeks EA treatment, the facial nerve examination of LSR disappeared, suggesting that the hyperexcitability of facial motor nucleus decreased, which was consistent with clinical symptoms and revealed a good prognosis. Hence, we thought that somatosensory inputs from the ipsilateral hand may *via* the medial lemniscus inhibit the facial inputs in the TN, so that it attenuated hyperexcitability of the facial motor nucleus and finally reduced the excitability of the facial nerve, then improved the symptoms.

For TMJ pain, the intensity of pain was assessed by VAS, which included scores from 0 (no pain at all) to 10 (strongest pain) (30). The level of the pain scores reduced to mild after 10 weeks of EA treatment, and without any related symptoms when treatments finished, suggesting that local acupuncture has a strong analgesic effect. The underlying mechanisms of acupuncture-induced pain relief were unclear, and several theories have been discussed, such as the endogenous opioid system (31), gate control theory (32), diffuse noxious inhibitory controls (DNIC) (33), and so on. Among these, the segmental mechanism is modulated by noxious stimulation at the local (34), while the DNIC can also be activated by stimulating in a distant area of the body (35). DNIC refers to the response from a noxious stimulus at a distance that attenuates pain from the second focal stimulus. Studies showed that patients with fibromyalgia (36), irritable bowel syndrome, or TMD disorder (37) have a reduced ability to inhibit pain, possibly due to the impaired DNIC. It has been proved that the analgesic effects are triggered by the activation of peripheral receptors carried by Aδ- and C-fibers (38), which is the same as the electrical acupuncture by activating the afferent nerve fibers innervating both the skin and muscles (39). So, we speculated that the segmental mechanism and DNIC may be the possible mechanism of acupuncture analgesia for TMJ pain.Overall, this is the first report of HFS and TMJ pain co-morbidity. The etiology and pathogenesis of this oral-facial disease co-morbidity are still unclear. However, it can be treated under the same principle from the perspective of meridian theory. Here, the local and distal acupoints were used for the treatment of facial and oral diseases, such as facial paralysis and trigeminal neuralgia (40–42). Studies have shown that acupuncture has a good effect on facial spasms and TMJ pain, respectively. Professor Chen used ipsilateral local acupoints combined with distal acupoints to treat primary and secondary hemifacial spasms, each lasting 30 min once in 2 days, for a total treatment of 12 days (43). Besides, another study on 127 HFS patients showed that local fire needle therapy with distal acupuncture also showed relieving on the symptoms of HFS (44). However, local stimulation, i.e., chronic electrical stimulation on the facial nerve induced hyperactivity of the facial nucleus (45), which may aggravate spasms. In terms of pain relief, local and distal stimulation with acupuncture (46), transcutaneous peripheral nerve stimulation (47), or vibratory stimulation (48) also showed a strong analgesic effect, but differed in stimulus parameters, such as intensity and frequency (47, 48). What's more, stimuli at distal or local acupoints can change the excitability in specific brain regions. For example, EA at LI 4 increased fMRI signal in the precentral gyrus (49) in healthy subjects, while EA at ST2 can induce interaction between face and hand representations of contralateral motor cortex in facial paralysis (50).

Compared with the other treatments (i.e., medical therapy, botulinum toxin type A and facial nerve decompression, and microvascular decompression), peripheral EA treatment showed few side effects and was more safe. It is easy to be accepted by patients in China. In this case, we applied ipsilateral distal acupoints (LI 4 and LU 10) to inhibit hyperexcitability of the spastic side, ipsilateral local acupoints (SI 18 and ST 7) for relieving TMJ pain, and improve blood circulation and other acupoints (EX-HN3 and GV 20) for regulating emotion, relieving anxiety, and stress. A 30-week EA treatment alleviated most of the symptoms of HFS and TMJ pain, but some symptoms might recur at the 6th follow-up, such as the frequency of eyelid spasms and pain in the TMJ. These recurrence symptoms do not affect a patient's daily life, but still disturb the patient and might cause mental stress. On the one hand, the instability of symptoms may be related to the complex pathogenesis of the disease itself, and on the other hand, long-time activities of facial expression muscles (such as smiling, chewing, talking, etc.), emotional disorders (51, 52), and insomnia may be important factors to induce the recurrence of the disease. Notably, the effects of EA lasted only a few months after treatment, revealing that EA is transient and reversible. Therefore, it needs a long-term EA treatment to maintain the effects.

It is important to note some limitations to the current findings of this case. First, the lack of radiological evidence, such as MRI, can be used as an auxiliary indicator to evaluate the changes in nerves, blood vessels, and joints in these two diseases. Second, the EA effect for relieving pain is obvious compared to attenuated spasms, which is essential to carry out further studies to verify the mechanisms of acupuncture in treating HFS and TMJ pain separately. Last, less was known about whether there was related interaction between the two types of facial disease. We are planning studies to address these limitations.

## Conclusion

In this study, we present a case of HFS and TMJ pain successfully treated with EA. EA with a frequency of 2 Hz and an intensity of ~1–2 mA showed effects of spasmolysis and analgesia for HFS and TMJ pain co-morbidity, which benefit for alleviating symptoms. Both distal and local acupoints play an important role in the treatment of co-morbidity. The potential mechanisms of EA on HFS and TMJ pain co-morbidity involved: (1) brain stem mechanism for distal acupuncture, where sensory input from the hand may inhibit facial afferent in the trigeminal nucleus *via* medial lemniscus; (2) segmental mechanism and DNIC for local and distal acupuncture analgesia.

## Data Availability Statement

The original contributions presented in the study are included in the article/supplementary material, further inquiries can be directed to the corresponding author.

## Ethics Statement

The studies involving human participants were reviewed and approved by Ethics Committee of the Guangdong Provincial Hospital of Chinese Medicine. The patients/participants provided their written informed consent to participate in this study. Written informed consent was obtained from the individual(s) for the publication of any potentially identifiable images or data included in this article.

## Author Contributions

J-hL and W-bF designed and drafted the manuscript. J-pH wrote the article and revised the manuscript. Z-mL and Q-wZ conducted the scale evaluation. W-tL and JZ conducted the EMG. SL and KL assisted in clinical treatment. All authors have read and agreed to the published version of the manuscript. All authors contributed to the article and approved the submitted version.

## Funding

This work was supported by the National Key Research and Development Program of China (grant number 2019YFC1709102), National Natural Science Foundation of China (grant numbers 81873381 and 82104979), Scientific Research Project of Traditional Chinese Medicine Bureau of Guangdong Province (grant numbers 20221169, 20221163, and 20201153), and Science and Technology Research Projects of Guangdong Provincial Hospital of Chinese Medicine (grant numbers YN2019ML12, YN2015QN19, and YN2020QN23).

## Conflict of Interest

The authors declare that the research was conducted in the absence of any commercial or financial relationships that could be construed as a potential conflict of interest.

## Publisher's Note

All claims expressed in this article are solely those of the authors and do not necessarily represent those of their affiliated organizations, or those of the publisher, the editors and the reviewers. Any product that may be evaluated in this article, or claim that may be made by its manufacturer, is not guaranteed or endorsed by the publisher.
