# Supplementary material for: Electroacupuncture on Hemifacial Spasm and Temporomandibular Joint Pain Co-Morbidity: A Case Report
Source: Front Neurol. 2022 Jun 28;13:931412. doi: 10.3389/fneur.2022.931412 (PMC9273903; doi:10.3389/fneur.2022.931412)
Supplement: Supplementary file 1 [file Table_1.DOCX]

**Table S1 Change in BR after EA treatment**

|  |  | R1 | |  | R2 | | | |
| --- | --- | --- | --- | --- | --- | --- | --- | --- |
|  |  | L (ms) | A (μV) |  | L (ms) | D (ms) | A (μV) | area (μV·ms) |
| nonspasm side | bef | 10.6 | 149.83 |  | 23.2 | 40.05 | 219.90 | 8807.57 |
|  | aft | 10.8 | 156.76 |  | 22.1 | 40.54 | 208.86 | 8466.25 |
| spasm  side | bef | 10.4 | 231.15 |  | 21.6 | 46.30 | 235.78 | 10916.71 |
|  | aft | 10.8 | 218.12 |  | 20.9 | 40.95 | 230.43 | 9436.34 |

L, latency; A, amplitude; D, duration
